# A novel figure-of-eight polymeric cerclage wiring technique for greater trochanter fixation in revision arthroplasty and conversion of failed proximal femur fracture fixations to total hip arthroplasty

**DOI:** 10.1186/s10195-026-00952-4

**Published:** 2026-07-13

**Authors:** Filippo Leggieri, Fernando N. Martín Cocilova, Gregorio Secci, Davide Stimolo, Luigi Zanna, Roberto Civinini, Matteo Innocenti

**Affiliations:** 1 Department of Orthopaedic Surgery, University of Florence, A.O.U. Careggi CTO, Largo Palagi 1, 50139 Florence, Italy; 2 Department of Orthopaedic Surgery, Santa Maria Annunziata Hospital, ASL Toscana Centro, Bagno a Ripoli, Italy

**Keywords:** Periprosthetic fracture, Greater trochanter, Polymeric cerclage wiring, Total hip arthroplasty, Revision surgery

## Abstract

**Introduction:**

Periprosthetic femoral fractures occur in 0.8–10% of primary total hip arthroplasties (THA) and up to 17.5% for revision THA (revTHA), with isolated greater trochanter (GT) fractures being the most predominant pattern. This study aimed to evaluate a novel figure-of-eight cerclage wiring technique for greater trochanter fixation in hip replacement cases.

**Methods:**

These retrospectively collected data included 36 hips (36 patients) undergoing revision THA or conversion of failed intertrochanteric fracture to THA with figure-of-eight cerclage wiring between April 2021 and June 2023. Patients under 18 years, with incomplete records, or lost to follow-up before 24 months were excluded. Primary outcomes included Trendelenburg sign, periprosthetic infections, and hip dislocation rates. Secondary outcomes evaluated THA stem subsidence and trochanteric migration using calibrated digital imaging. Statistical analysis included descriptive statistics, independent *t*-tests, linear regression, and correlation analyses with significance set at *p* < 0.05.

**Results:**

Mean follow-up was 404.76 ± 22.51 days. Patient demographics showed mean age 76.5 ± 12.3 years, body mass index (BMI) 25.8 ± 4.5 kg/m^2^, with 73.8% female patients. At final follow-up, one patient (2.8%) experienced post-surgical infection, one patient (2.8%) had dislocation, and three patients (8.3%) exhibited positive Trendelenburg sign. Mean trochanteric migration was 4.01 mm at 12 months (range 0.0–30.4 mm), with one clinically significant case (30.4 mm migration). Mean stem subsidence was 1.3 ± 2.7 mm (range 0.0–16.4 mm), with one clinically significant case (16.4 mm subsidence). No statistically significant differences were found between posterior-lateral and anterior approaches for migration (*p* = 0.276) or subsidence (*p* = 0.324). Linear regression analysis revealed no significant predictors of migration or subsidence among age, BMI, surgical approach, or treatment type.

**Conclusions:**

The figure-of-eight cerclage wiring technique with polymeric cerclages demonstrates promising results for greater trochanter fixation with low complication rates and adequate stability across different surgical approaches and patient populations.

*Level of Evidence* IV.

## Introduction

The incidence of total hip arthroplasty (THA) is increasing, with projections suggesting continued growth in the coming decade [1, 2]. Alongside this surge in procedural volume comes an inevitable rise in associated complications. Periprosthetic femoral fractures represent a significant concern, occurring in 0.8–10% of primary THAs [1, 2]. Among the periprosthetic fractures whose incidence rate account for 3.5% at 20 years for primary THA and 17.5% for revision THA (revTHA), isolated greater trochanter (GT) fractures emerge as the most predominant pattern [3, 4].

The majority of these fractures (83.7%) in revTHA occur during femoral canal preparation or component placement [4] and often result in prolonged recovery, compromised functional outcomes, and high revision rates [5–7].

Similarly, failures of proximal femur fixation are also frequently observed in the elderly population presenting with complex and unstable intertrochanteric fractures [8, 9]. In such cases, during conversion to THA using a revision stem, one of the main challenges is the need to reattach the greater trochanter, which is often already displaced or fractured intraoperatively owing to the bone weakness related to the failure of the previous fixation.

Management strategies of intraoperative trochanteric fractures or trochanteric bone displacement due to previous proximal femur fixation failures vary from conservative approaches to various fixation techniques. While some stable, undisplaced fractures may represent indications to nonoperative treatment [6, 10, 11], the displacing forces of the abductor musculature often necessitate surgical intervention. Fixation options include cerclage wires, cables, screws, and plating systems, with selection dependent on fracture pattern and surgeon preference [6, 10–13]. Literature reporting on the outcomes of these various fixation options is heterogeneous, and no fixation method has proven superiority.

Figure-of-eight cerclage wiring has demonstrated favorable clinical outcomes with less patient discomfort and fewer hardware-related complications across various orthopedic applications [14–19], yet, to date no literature exists on its application in preventing or treating periprosthetic hip fractures.

The aim of this study was to evaluate the clinical and radiographic outcomes of a novel figure-of-eight cerclage wiring technique with a polymeric cerclage for greater trochanter fixation in THA and revision THA, specifically assessing postoperative complications (infection, dislocation, hardware-related issues), abductor function (Trendelenburg sign), and radiographic stability (femoral stem subsidence and trochanter migration). The hypothesis was that the novel technique would provide reliable fixation with low complication rates and preserved function, representing a viable alternative to existing trochanteric fixation methods.

## Materials and methods

### Study design and population

This retrospective cohort study was conducted at a single institution between April 2021 and June 2023. Data collection occurred between December 2024 and March 2025. Written informed consent was obtained from all participants. Patients undergoing revision total hip arthroplasty (THA) or conversion of failed intertrochanteric fracture to THA with figure-of-eight cerclage wiring with a polymeric cerclage for greater trochanter (GT) fixation were included. Exclusion criteria included patients under 18 years of age, those with incomplete medical records, and those lost to follow-up before the minimum follow-up period of 24 months. The study was conducted in accordance with the Declaration of Helsinki and it was conducted following the Strengthening the Reporting of Observational Studies in Epidemiology (STROBE) guidelines [20]. The screening of medical records identified 42 patients. However, four patients were excluded owing to missing the required 6-month outpatient visit and X-rays, and two additional patients did not provide consent to participate in the study.

### Surgical technique

All procedures were performed by two surgeons, and the surgical approach, whether posterior-lateral (PL) or anterior base muscle sparing (ABMS), was determined by surgeon preference on the basis of case-specific factors. For the figure-of-eight cerclage wiring technique, the same polymeric cerclage wiring (SuperCable^®^, Kinamed Inc., Camarillo, CA) was used.

After preparation of the femoral canal and insertion of the femoral component, the cerclage wire was passed around the proximal femur in a figure-of-eight configuration. The configuration can be adapted in different configurations on the basis of the presence or absence of the lesser trochanter.

#### Classic configuration (Fig. 1)

**Fig. 1:**
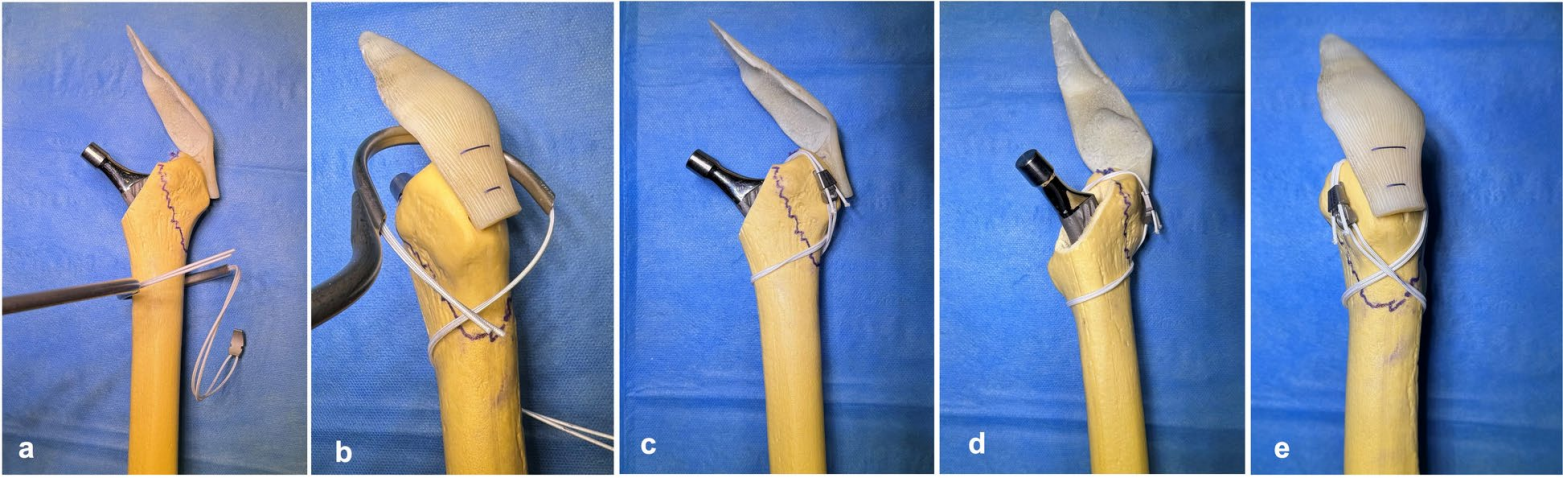
Classic figure-of-eight cerclage configuration. Greater trochanter fracture was marked with blue ink. **a** The cable was passed around the proximal third of the femur just below the lesser trochanter using a dedicated loop cerclage instrument. **b** Using a Deschamps passer, the longer cerclage end was passed from lateral to medial in a loop around the gluteus medius and through the pouch between the gluteus medius and the apex of the greater trochanter. **c** Final aspect of the cerclage configuration from the anterior view: the metallic clip of the cerclage was positioned in the anterolateral part of the proximal femur. **d** Medial view of the final aspect of the cerclage configuration: the lesser trochanter acts as an anchor for the cable which passes below it. **e** Lateral view of the cerclage configuration from the lateral view

This approach was used when the lesser trochanter was structurally and functionally intact. Using a dedicated loop cerclage instrument, the cable was passed around the proximal third of the femur just below the lesser trochanter, which acts as an anchor for the construct. The two ends of the cerclage are then crossed over the lateral aspect of the greater trochanter. The shorter end—bearing the clip for final locking—was intentionally kept shorter, allowing it to be reconnected with the longer extremity after the latter has been looped around the greater trochanter.

The insertion of the gluteus medius (GM) on the greater trochanter was then identified, and the GM fan was isolated. Using a Deschamps passer, the longer cerclage end was passed from lateral to medial in a loop around the GM and through the pouch between the GM and the apex of the greater trochanter. Once the loop was completed, the two cerclage ends were reconnected and properly tensioned at the anterolateral portion of the trochanteric mass until the construct was deemed stable. During tensioning, it is advisable not to exceed the “low” setting indicated on the tensioning device, as a higher force may cause splitting of the greater trochanter or avulsion of the gluteus medius insertion.

#### Lesser trochanter detachment or fracture

In cases of an associated avulsion or a fracture of the lesser trochanter, two scenarios are possible.

**Fig. 2:**
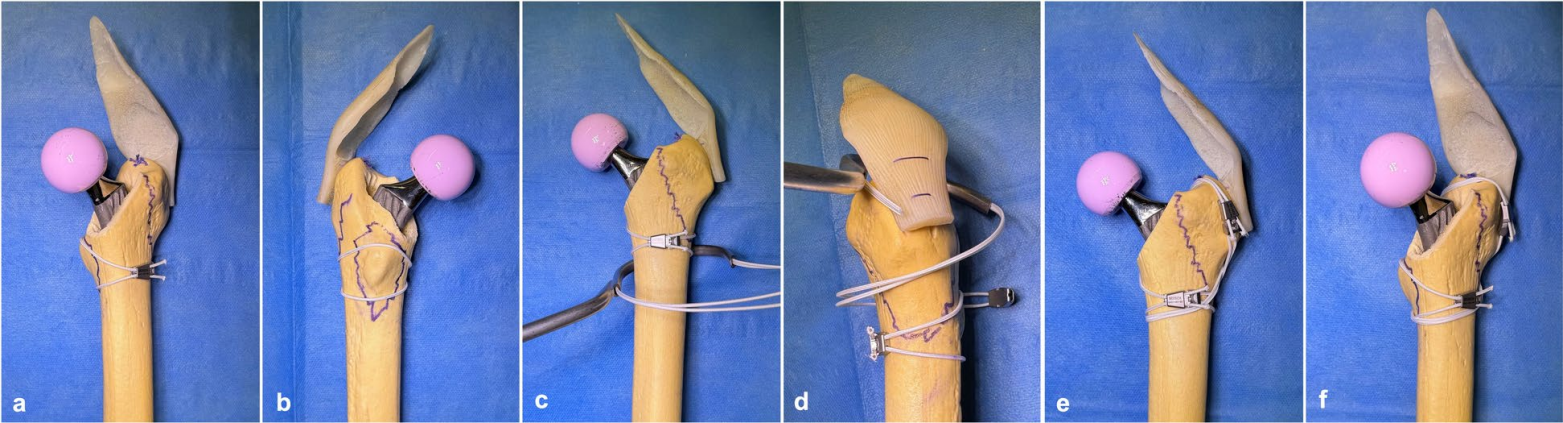
Configuration in case of lesser trochanter detachment or fracture with feasible fixation. Greater and lesser trochanter fractures were marked with blue ink. **a** Sling fixation technique, anterior view. **b** Sling fixation technique, medial view. **c** The cable was passed around the proximal third of the femur just below the lesser trochanter as in the classic configuration. **d** The longer cerclage end was passed from lateral to medial in a loop around the gluteus medius as in the classic configuration. **e** Anterior view of the final aspect of the cerclage configuration: the cable of the sling fixation of the lesser trochanter acts as an anchor for the cable which passes below it. **f** Medial view of the final aspect of the cerclage configuration

When the anatomical reduction and fixation of the lesser trochanter are feasible, a sling fixation technique is then used firstly [21], followed by the same novel aforementioned technique using a total of two cerclages (Fig. 2).

**Fig. 3:**
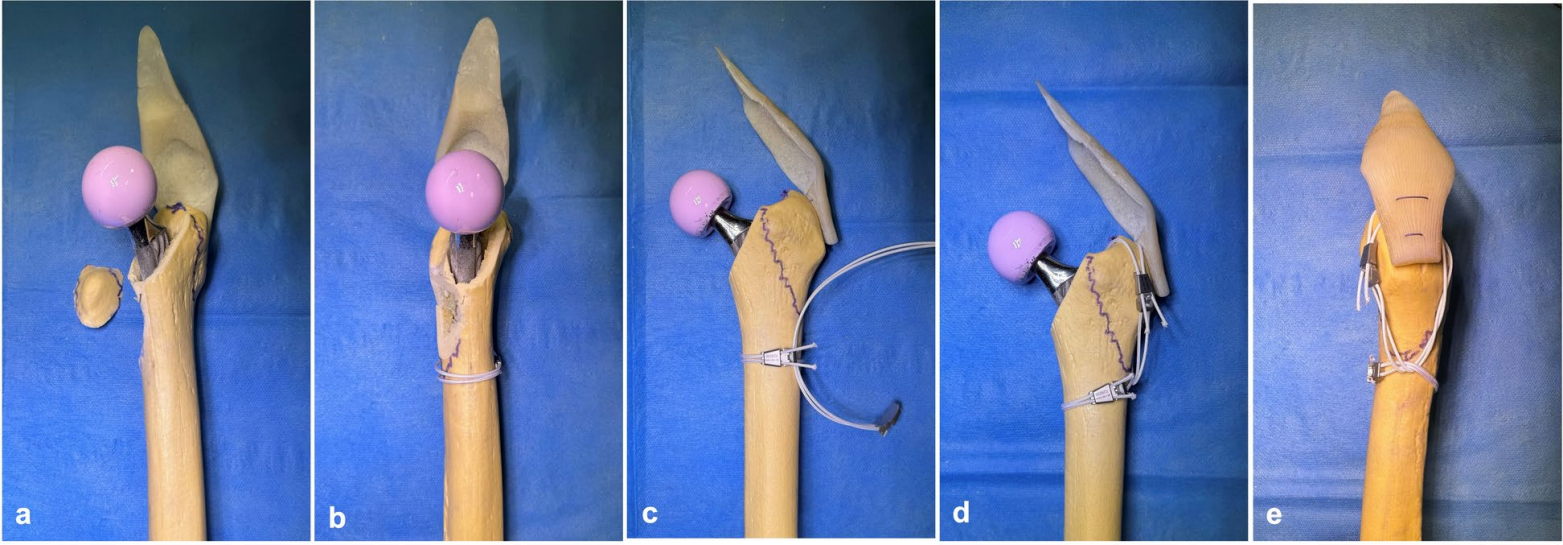
First configuration in case of lesser trochanter detachment or fracture without feasible fixation. Greater trochanter fracture was marked with blue ink; lesser trochanter is detached. **a** Detachment of the lesser trochanter. **b** A primary loop cerclage can pass around the most structurally intact portion of the proximal femoral diaphysis. **c** Before tensioning this first cerclage, a second cerclage is passed around it on the lateral side in a sling-like fashion. **d** The second cerclage is subsequently passed around the trochanteric region. **e** Figure-of-eight configuration created by the two cerclages

**Fig. 4:**
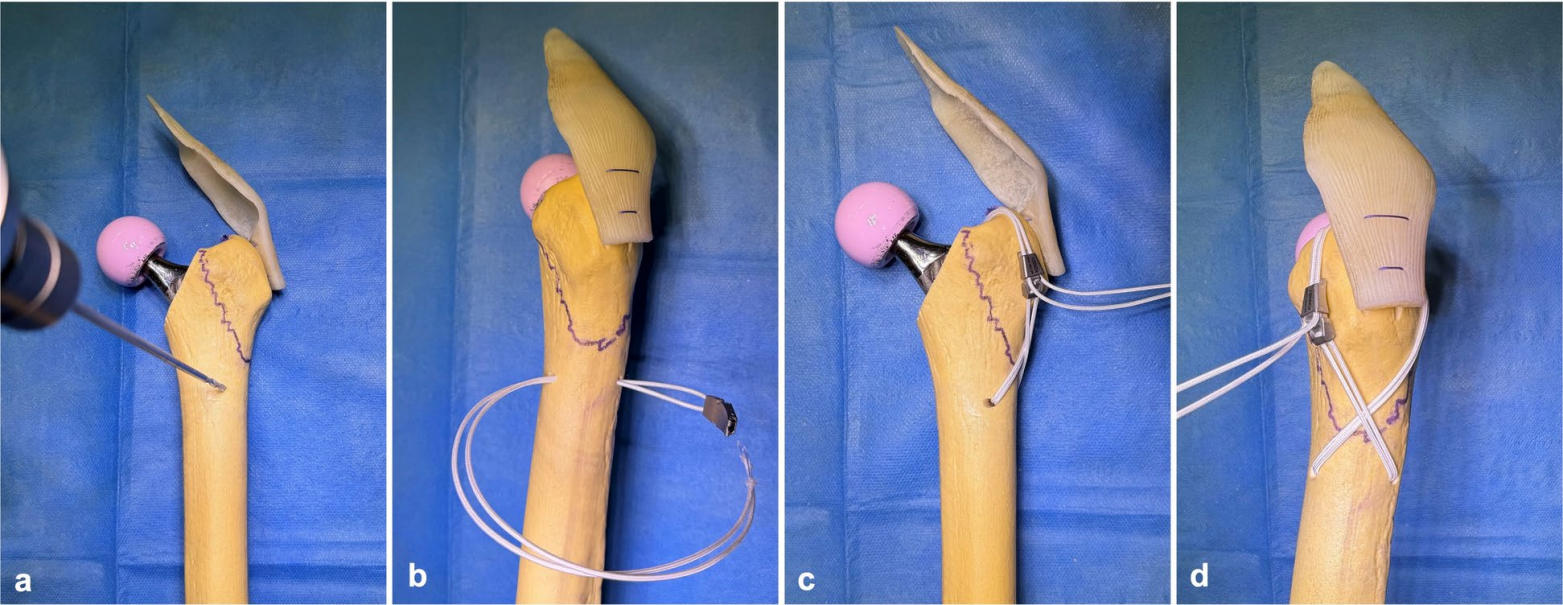
Second configuration in case of lesser trochanter detachment or fracture without feasible fixation. Greater trochanter fracture was marked with blue ink; lesser trochanter is detached. **a** A 3.5-mm hole drilled in the lateral cortex of the proximal femoral diaphysis. **b** The cerclage is passed through the hole. **c** The cerclage is passed around the grater trochanter to create the classic figure-of-eight configuration. **d** Lateral view of the final aspect of the cerclage configuration

Contrarily, the reconstruction of the lesser trochanter may not be viable following a complete absence of the lesser trochanter that may occur owing to osteolysis, comminuted fracture, or proximal migration following failed intertrochanteric fracture treatment. In such cases, there are two techniques to achieve the figure-of-eight configuration. On the one hand, a primary loop cerclage can pass around the most structurally intact portion of the proximal femoral diaphysis. Before tensioning this first cerclage, a second cerclage is passed around it on the lateral side in a sling-like fashion. The first cerclage is then tensioned to firmly anchor the second one, which is subsequently passed around the trochanteric region in the previously described figure-of-eight configuration using a total of two cerclages (Fig. 3). On the other hand, a hole can be drilled in the lateral cortex of the proximal femoral diaphysis. Through this eyelet, a cerclage is passed and then used to create the classic figure-of-eight configuration as previously described (Fig. 4). For each scenario, once the cerclage has been applied, rotational, adduction and abduction stress tests are performed to assess the stability of the proximal bone fragment.

### Data collection

Patient demographic data including age, sex, body mass index (BMI), side, and diagnosis at admission were extracted from electronic medical records. Surgical data collected included surgical approach, surgical intervention modality, and reason for cerclage application. Clinical outcomes were assessed at regular follow-up visits at 1 months, 6 months, and 12 months. Anteroposterior pelvic and anteroposterior and lateral affected hip X-rays were obtained preoperatively, immediately postoperatively, and at each follow-up visit. All radiographic measurements were performed by two independent observers blinded to patient outcomes.

### Outcome measure

The first endpoint of this study was to measure positive Trendelenburg sign, peri-implant and periprosthetic join infections, and hip dislocation rates. Peri-implant and periprosthetic join infection was defined according to the International Consensus Meeting on Musculoskeletal Infection criteria [22].

Secondary outcomes included the evaluation of stem subsidence (mm) and GT migration (mm). Radiographic criteria established by Engh et al. [23] were used to assess femoral stem subsidence. The stem’s height within the proximal femur was measured by drawing a vertical line along its lateral longitudinal axis. Two additional horizontal lines were then drawn perpendicular to this axis: one passing through the superior tip of the greater trochanter and the other through the stem’s shoulder. Subsidence was evaluated by measuring changes in the distance between these two reference points. A change greater than 10 mm was considered clinically significant [24]. GT migration was defined as the vertical displacement of the greater trochanter from its anatomical position relative to its own apex, following a previously validated protocol [25]. A change greater than 20 mm of GT migration was considered clinically significant [26]. Measurements were performed using calibrated digital imaging software (TraumaCad^®^, Brainlab AG, Munich, Germany).

### Data analysis

No missing data were reported. Descriptive statistics were calculated for all study variables as means and medians, standard deviations (SD), interquartile ranges (IQR), and ranges (minimum to maximum values). Categorical data were presented as frequencies and percentages.

The reliability of measurements was assessed with intraclass correlation coefficients (ICC) for radiographic evaluations at 1, 6, and 12 months, with values > 0.9 indicating excellent reliability. To compare outcomes between different approaches, independent samples *t*-tests with mean differences were performed and Cohen’s *d* was used to quantify effect sizes. Linear regression analyses were conducted to identify potential predictors of GT migration and implant subsidence. Independent variables included age, body mass index (BMI), surgical approach, and treatment type. Beta coefficients were calculated to determine the strength and direction of associations. Pearson correlation coefficients were calculated to assess relationships between continuous variables (age, BMI, migration, subsidence) and point-biserial correlations for associations between continuous and binary variables (presence of Trendelenburg sign, infection, dislocation). The level of statistical significance was set at *p* < 0.05. Statistical analysis was performed using SPSS software (IBM, Armonk, New York).

## Results

According to inclusion and exclusion criteria, the final population included 36 hips (36 individuals) with a mean follow-up of 404.76 ± 22.51 (range 359–440) days. Six patients were excluded owing to an insufficient follow-up time. The intraclass correlation coefficient (ICC) was found to be 0.94 (95% confidence interval (CI) 0.91–0.97) at 1 month, 0.94 (95% CI 0.91–0.96) at 6 months, and 0.93 (95% CI 0.90–0.95) at 12 months, indicating excellent reliability. Baselines characteristics are presented in Table 1. Table 2 presents data about the frequencies of different diagnosis and treatment modalities among the included population.

**Table 1:** Baseline characteristics of the included population

Variable	Mean ± SD	Median [IQR]	Range
Age (years)	76.5 ± 12.3	77.5 [70.3–88.3]	48.0–96.0
BMI (kg/m^2^)	25.8 ± 4.5	24.9 [23.1–26.2]	19.5–42.5
Female sex [*n* (%)]	31 (73.8%)		
Left side [*n* (%)]	19 (45.2%)		
PL approach [*n* (%)]	23 (54.8%)		
ABMS approach [*n* (%)]	19 (42.5%)		
Maximum migration (mm)	4.0 ± 6.2	2.0 [0.3–3.5]	0.0–30.4
Maximum subsidence (mm)	1.3 ± 2.7	0.1 [0.0–2.1]	0.0–16.4

**Table 2:** Baselines about diagnosis and surgical intervention modalities among the included population

Diagnosis	*n*	(%)	Treatment	*n*	(%)
Cephalomedullary nail failure	20	55.5	Nail removal + THA	20	55.5
DHS failure	4	11.1	Plate removal + THA	4	11.1
PJI	3	8.3	Two-stage revision THA	3	8.3
Failed THA	9	25	Revision THA	9	25

At the final follow up, among the 36 patients analyzed, one patient (2.8%) experienced a post-surgical infection and one patient (2.8%) had a dislocation. In addition, three patients (8.3%) exhibited a positive Trendelenburg sign.

The subsidence trend demonstrated that maximum subsidence was generally reached at the 1-month follow-up, except for one case in which a clinically significant subsidence occurred at 6 months (maximum subsidence: 16.4 mm, maximum trochanteric migration: 0 mm). No other complications were observed in this patient throughout the follow-up period, and no subsequent reintervention was performed owing to the high anesthetic risk and the absence of progressive subsidence beyond the 6-month visit. GT migration increased over time, with a mean of 1.87 mm (range 0.00–16.72 mm, IQR 0.11–1.69 mm) at 1 month, 3.56 mm (range 0.00–25.40 mm, IQR 0.25–3.39 mm) at 6 months, and 4.01 mm (range 0.00–30.40 mm, IQR 0.30–3.53 mm) at 12 months. One case of GT migration was clinically significant (maximum migration = 30.4 mm, maximum subsidence = 2.3 mm) in a female patient of 75 years old treated owing to cephalomedullary proximal femur nail failure who underwent hardware removal and THA surgery. After appropriate informed consent, the patient decided not to receive revision surgery for minimal subjective impairment with no sign of uncompensated Trendelenburg but a subtle compensated Trendelenburg sign.

A comparison of the PL and ABMS approaches showed no statistically significant differences in migration or subsidence. The mean migration was 4.93 mm ± 7.08 in the PL group and 2.89 mm ± 4.83 in the ABMS group, with a mean difference of 2.04 mm (95% CI 1.70–5.78, *p* = 0.276) and a small effect size (Cohen’s *d* = 0.33). Similarly, mean subsidence was 1.69 mm ± 3.48 in the PL group and 0.89 mm ± 1.36 in the ABMS group, with a mean difference of 0.79 mm (95% CI 0.82–2.41, *p* = 0.324) and an effect size of 0.29.

The linear regression analysis assessing factors associated with GT migration and implant subsidence found no statistically significant associations for any of the examined variables, including age, BMI, surgical approach, or treatment type (Table 3).

**Table 3:** Regression analysis for migration and subsidence

	Migration (mm) coefficient (95% CI)	*p*-value	Subsidence (mm) coefficient (95% CI)	*p*-value
Age	0.04 (−0.15, 0.22)	0.699	0.02 (−0.06, 0.11)	0.573
BMI	− 0.00 (−0.49, 0.48)	0.984	0.05 (−0.17, 0.27)	0.647
PL approach	− 1.73 (−6.08, 2.63)	0.426	− 0.86 (−2.81, 1.10)	0.379
Revision THA	3.72 (−16.37, 23.82)	0.287	− 1.64(−10.67, 7.38)	0.714
Primary THA	− 3.25 (−7.84, 1.34)	0.159	0.89 (−1.17, 2.95)	0.388
ORIF with plating	− 0.81 (−14.53, 12.92)	0.906	2.58 (−3.59, 8.75)	0.402
Hardware removal + IM	− 1.14 (−14.96, 12.69)	0.868	− 0.76 (−6.97, 5.45)	0.805
Hemiarthroplasty	− 3.96 (−11.67, 3.76)	0.305	− 0.17 (−3.64, 3.29)	0.921

The correlation matrix revealed no strong nor significant linear relationships between age, BMI, migration, or subsidence (*p* < 0.05). Migration and subsidence were almost uncorrelated (*r* = −0.025, *p* = 0.874). The association between migration and Trendelenburg sign showed a weak and nonsignificant positive correlation (*r* = 0.22, *p* = 0.1545). Similarly, migration had a weak positive correlation with infection (*r* = 0.16, *p* = 0.316) and a weak negative correlation with dislocation (*r* =  −0.009, *p* = 0.55). For subsidence, no meaningful correlations were observed with Trendelenburg sign (*r* =  −0.11, *p* = 0.491), infection (*r* = 0.09, *p* = 0.567), or dislocation (*r *=  −0.08, *p* = 0.628) (Table 4).

**Table 4:** Correlation between migration and subsidence with clinical outcomes

	Coefficient	*p*-value
Migration and Trendelenburg	0.224	0.155
Migration and infection	0.158	0.317
Migration and dislocation	−0.095	0.551
Subsidence and Trendelenburg	−0.109	0.492
Subsidence and infection	0.091	0.567
Subsidence and dislocation	−0.077	0.629

## Discussion

**Fig. 5:**
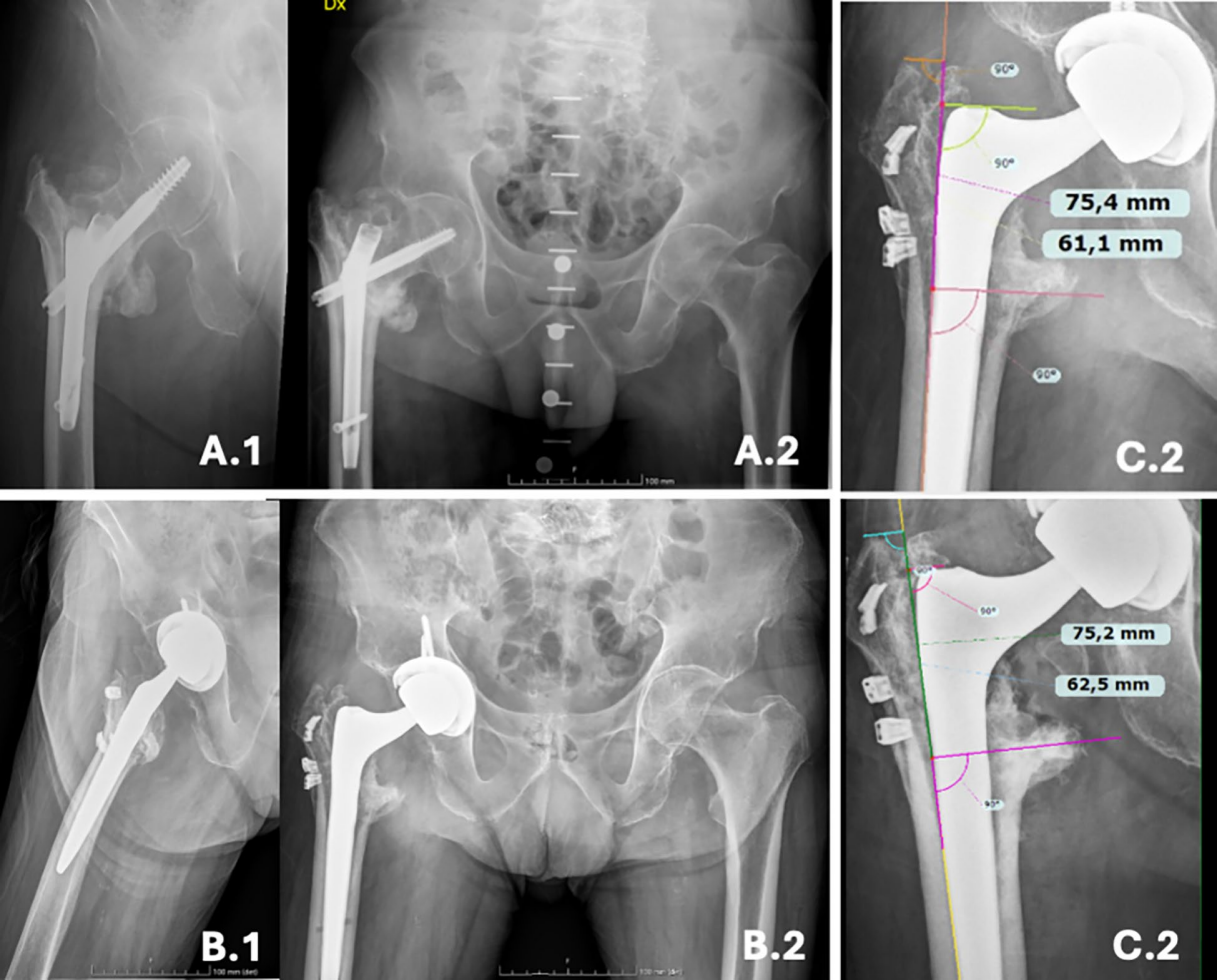
Conversion to revision total hip arthroplasty following a cephalomedullary nail breakage, with figure-of-eight polymeric cerclage wiring of the greater trochanter and proximal femur. **a**.**1** Preoperative AP view radiograph of the right hip showing the nail breakage. **a**.**2** Preoperative AP view radiograph of the pelvis showing. **b**.**1** Early term postoperative axillary view radiograph of the right hip showing the nail breakage. **b**.**2** The 1-month postoperative AP-view radiograph of the pelvis showing the nail breakage. **c**.**1** The 1-month postoperative AP-view radiograph of the right hip showing trochanter migration and stem subsidence assessment. **c**.**2** The 1-year postoperative AP-view radiograph of the right hip showing trochanter migration and stem subsidence assessment. AP, anteroposterior

This study investigated the application of a novel figure-of-eight cerclage wiring technique for GT fixation in THA/revTHA, specifically in cases of failed previous arthroplasty or proximal femur fracture fixation. Our findings demonstrated that this technique offers promising clinical and radiographic results, suggesting it may be a valuable addition to the surgical armamentarium for trochanteric fixation (Fig. 5).

In our cohort of 36 hips, we observed a low overall complication rate: one case of infection (2.8%), one dislocation (2.8%), and three cases (8.3%) of positive Trendelenburg sign. Radiographically, the figure-of-eight cerclage configuration provided adequate stability. While some trochanteric migration was observed (mean 4.01 mm at 12 months), this did not lead to significant clinical impairment, as shown by the low Trendelenburg rate and preserved abductor function in most patients. Importantly, there was no statistically significant correlation between the fixation technique and negative outcomes across demographic or surgical variables. Linear regression analysis showed that factors such as age, BMI, surgical approach, or treatment type did not significantly predict either trochanteric migration or femoral stem subsidence. In addition, a comparison between PL and ABMS approaches revealed no significant differences in either migration or subsidence, indicating that the effectiveness of the figure-of-eight technique is not approach dependent.

In terms of fixation methodology, various techniques for GT fracture fixation using both wires or cables have been described in literature [6, 11, 26, 27]. Both monofilament wires and multifilament cables offer advantages but also carry limitations. Wires can be challenging to tighten adequately and are prone to breakage, especially when kinked [28–41]. Cables were developed with superior fatigue resistance and tensile strength, yet clinical data show similar breakage rates to wires [6, 11]. Complications with both include loosening, breakage, and migration of the hardware [6, 11]. In contrast to our findings—which included only one case of significant trochanteric migration—wire fixation in literature shows nonunion rates from 0% to 28%, with higher rates among males, rheumatoid arthritis patients, and in revision settings [42].

The use of non-metallic fixation materials in tension-band constructs has gained increasing support across various orthopedic applications. Non-absorbable braided polyblend sutures have demonstrated adequate mechanical stability with favorable clinical outcomes and significantly reduced hardware-related complications in the treatment of patellar fractures, where metallic constructs are associated with symptomatic hardware requiring reoperation in up to 60% of cases [43, 44]. Similar findings have been reported for displaced olecranon fractures, where tension band suture fixation with FiberWire showed no revision surgeries at 4-year follow-up and a high union rate [45].

Compared with cables, wires are associated with higher technical variability, while cables raise concerns regarding metallic debris from fraying, potentially leading to osteolysis and increased acetabular loosening [10] [46, 47]. Furthermore, osseous ingrowth into cables can complicate implant removal during revision procedures. Although simpler wiring techniques show favorable trends in outcomes, they have not been shown to be consistently appropriate for technically challenging indications such as revision THA and prior trochanteric nonunion. Among current systems, cable-grip fixation is frequently reported in both primary and revision settings [31]. These systems employ claw designs that help secure GT fragments after reduction. Sequential cable tightening enhances rigidity [31] and can support allograft use to promote healing [48]. However, like cerclage cables, cable-grip systems are vulnerable to hardware migration and breakage, though they withstand loads approximately 1.5 times greater than cables alone [10]. Proper surgical technique—including maintaining an intact medial cortex distal to the lesser trochanter and avoiding direct contact with the femoral stem—is critical to reducing early failure [6]. Larger cable diameters (2.0 mm versus 1.6 mm) significantly lower early failure rates [39]. For comminuted trochanteric fractures or those with severe osteolysis, plate fixation may be warranted. Common options include claw plates with proximal spikes and locking plates with multiple screws [49–51]. Biomechanical data support the superior stability of plating over cable fixation alone [52]. While claw and locking plates have long been used in trauma, their application in trochanteric reattachment has only gained attention in the past decade [10]. These plates are typically employed in cases of established nonunion with poor bone stock owing to their ability to achieve stable fixation with unicortical screws [53]. However, results are controversial—some studies report clinical benefits with claw plates [49, 51], while others show high rates of nonunion or fragment migration [49, 51]. Despite promising biomechanical stability, current literature suggests relatively high reoperation rates with claw and locking plates [10].

In cases of intraoperative GT fractures during THA, several approaches are reported. If the fracture is intraoperatively diagnosed, immediate fixation with cables, wires, or sutures can be effective [7]. If the fracture is only diagnosed postoperatively and does not result in instability, pain, or significant displacement (> 2 cm), non-operative management may be considered [54]. Series by Hartford et al. [5], Foissey et al. [55], and Brun et al. [56] indicate no clear differences in outcomes between operatively and non-operatively treated intraoperative fractures.

Postoperative GT fractures may occur acutely owing to unrecognized intraoperative events or later owing to falls or trauma. Systematic reviews of isolated GT fractures not associated with a THA often advocate for conservative treatment regardless of fragment displacement [57, 58]. Despite the additional biomechanical challenges posed by a prosthesis, these non-operative principles may still apply to acute postoperative GT fractures. However, postoperative GT fractures may present unique challenges owing to implant-related bone loss due to stress shielding from distally fixed stems, and debris-induced osteolysis complicates management. Evidence supports both nonoperative treatment [59, 60] and surgical intervention using wire fixation with or without bone grafting [61].

The surgical management of GT nonunion in THA patients has yielded variable results. Claw plates demonstrate union rates of 70.8%, though significant pain and limp are frequently reported [62]. Dual plates [33] and standard locking plates [63] showed high union rates (87% and 92.3%, respectively) but are associated with implant-related complications necessitating hardware removal in up to 20% of cases. Patients with persistent nonunion generally exhibit poorer functional outcomes, with Harris Hip Scores about 19 points lower than those who achieve union [63].

Although no comparative studies exist on surgical versus nonoperative treatment for established GT nonunion in THA patients, fragment excision has been attempted with mixed outcomes in symptomatic cases after failed surgery [64]. Finally, GT fractures during revision hip arthroplasty pose unique challenges owing to compromised bone stock, difficult reconstruction requirements, and poor soft tissues status, with most literature focusing on surgical fixation outcomes while emphasizing the importance of considering specialized bearing surfaces like dual mobility or constrained liners to address potential stability concerns [6, 10, 11].

When comparing our outcomes to existing literature, the figure-of-eight cerclage technique shows favorable results. Our 8.3% Trendelenburg rate compares well with reported rates: 5.42% (95% CI 3.63–7.22%) for wires, 13.23% (95% CI 0–33.66%) for cables, 24.80% (95% CI 0–49.59) for cable grip/plate, and 18.72% (95% CI 5.34–32.11) for claw/locking plate) [10]. Our dislocation rate of 2.8% is also comparable or slightly lower than the rates seen with cable grip systems (2.17, 95% CI 0–4.53 dislocations) and claw/plate fixation (5.35, 95% CI 1.56–9.15 dislocations) [10]. Notably, the mean migration in our series (4.01 mm) is substantially lower than the 7.31 mm reported with traditional cerclage wiring techniques (7.31 mm, 95% CI 5.81–8.81) [10].

While the absence of a control group precludes any direct comparison with alternative fixation techniques, the outcomes observed in this cohort are consistent with those reported in literature for established methods, supporting the technical feasibility of the proposed technique and warranting further comparative investigation. The consistent performance across patient subgroups and surgical approaches further underscores its broad applicability.

Nevertheless, several limitations must be acknowledged. The study’s retrospective design, single-institution setting, and limited sample size constrain generalizability. In addition, the absence of a control group using alternative fixation techniques limits direct comparison. Although definitive comparisons between implants cannot be made without a randomized trial, it remains uncertain whether the superior mechanical properties of cables translate into improved clinical outcomes. Furthermore, the relatively short follow-up period may not be sufficient to capture late complications such as delayed trochanteric migration, nonunion, or fixation failure. Longer-term follow-up studies are warranted to fully characterize the durability of the proposed technique. The heterogeneity of the cohort may have influenced the results, as the included cases differed in baseline bone quality, bone defect extent, and abductor apparatus integrity among the other parameters. A formal subgroup analysis was not feasible given the limited sample size. Higher-quality comparative studies, including randomized controlled trials (RCTs), are warranted to further define the role of this technique. Furthermore, the absence of validated functional outcome scores represents a limitation. Our findings support the hypothesis that the figure-of-eight cerclage wiring technique offers a reliable, minimally invasive alternative that maintains stability and function, even in complex revision setting.

## Conclusions

The figure-of-eight cerclage wiring technique with polymeric cerclage appears to be a promising method for greater trochanter fixation in revTHA or THA performed after failures of intertrochanteric fixation, offering adequate stability with a low complication profile. The technique’s versatility across different surgical approaches and patient populations represents a potential advantage in the management of trochanteric fixation challenges in both primary and revision hip arthroplasty.

## Data Availability

The datasets used and/or analyzed during the current study are available from the corresponding author on request.
